# The efficacy of preopoerative instruction in reducing anxiety following gyneoncological surgery: a case control study

**DOI:** 10.1186/1477-7819-9-38

**Published:** 2011-04-08

**Authors:** Gul Pinar, Ayten Kurt, Tayfun Gungor

**Affiliations:** 1 Başkent University Health Sciences Faculty, Nursing and Healthcare Services Department, Eskisehir Yolu, 20. km. Balica Campus, Cayyolu/Ankara- Turkey; 2 Zekai Tahir Burak Gynecology Training and Research Hospital, Gundogdu Mah. Karacabey Sok. Hamamonu, Turkey

## Abstract

**Background:**

This is a quasi-experimental case control research focusing on the impact of systematic preoperative instruction on the level of postoperative anxiety in gyneoncologic patients. The population studied consists of the gyneoncologic surgery patients admitted to the Gynecologic Oncology Service at Zekai Tahir Burak Gynecology Training and Research Hospital from May to September 2010.

**Patients and methods:**

Through a random sampling, 60 patients were recruited in each group. The study group was given a systematic preoperative instruction while the control group was given routine nursing care. Patients were interviewed in the postoperative period and anxiety was measured. The data-collecting tool consisted of the Individual Information Form and the State-Trait Anxiety Inventory. The collected data were analyzed by using the SPSS Program to find the frequency, the percentage, the mean and the standard variables, and the hypothesis was tested with Chi-square, variance, and t-independent test.

**Results:**

It was found that the incidence rates from the post-operative anxiety score of the study group were lower than those of the control group (p < .05). The results of this research demonstrated that gyneoncologic surgery patients who were given systematic preoperative instruction felt less anxious than the ones who were given merely a routine nursing care.

**Conclusions:**

Results of this study suggest that preoperative instruction programs aiming at informing gyneoncologic surgery patients at the preoperative stage should be organized in hospitals and have an essential role.

## 1. Background

Anxiety is an individual experience and it is a concept that is difficult to describe with words. No matter how major or minor an operation is, it tends to raise a certain level of anxiety in every patient [1]. Hospitalization for surgical procedure can be experienced as a threat or stressor and may produce anxiety in patients. Anxiety occurs in the preoperative phase as the patients anticipate an unknown event with potential pain and changes in body image, as well as increased dependency on family and other life changes [2].

Although some of the patients know in advance that they are going to be treated by an operation, they cannot help feeling worried, anxious, and nervous about the upcoming surgical treatment. The patients diagnosed with gynecological cancer often respond by wanting everything possible done to remove the cancer. Anxiety is one of the most frequent and widespread psychosocial problems seen particularly in gynecologic cancers [1]. Especially hysterectomy is a surgical procedure that significantly affects the quality in which the operated person views herself, lowers self-esteem and brings about changes in the quality of life [3,4]. While a postmenopausal woman, who has completed her reproductive life, may view a hysterectomy as the removal of an organ that has "turned bad," a young woman may have a very different viewpoint [5]. They want the doctors and the nurses to explain to them the details of their ailment, the operation, and the procedure of the pre and postoperative self - practices [6-8].

Often, the information provided for the patients does not cover the necessary medical regimen which will help them when they have to face the problems and solve them properly. Giving systematic advice and information is very rare. Actually, the health personnel should give patients information about what they will have to face on the operation day, such as the characteristics of the operating theatre, and the medical procedures before they fall asleep because of the effect of anesthesia [9,10]. The patients who are given the systematic instruction will obtain right and sufficient information, and develop a positive attitude. They will also be willing to follow the medical practices. When anxiety diminishes, the negative mental and emotional states, such as irritation, aggression, lack of concentration, and depression will also reduce. It can help patients to recover more rapidly and reduce the length of time of hospital stay since giving them appropriate knowledge can make them change their beliefs and behaviors [1,9].

### 1.1. Objective

The aim of this study was to examine the effect of preoperative instruction on anxiety level after gyneoncologic surgery. The sociodemographic and medical characteristics which are thought to have an impact on anxiety were addressed, as well.

### 1.2. Hypotheses

The level of postoperative anxiety of the study group who were given a systematic preoperative instruction were found lower than those of the control group who were routinely treated.

## 2. Methods

### 2.1. Type of the Study

This is a quasi-experimental research based on one study group and one control group and it focuses on the study of the impact of systematic preoperative instruction on the level of post-operation anxiety of gyneoncologic patients.

### 2.2 Time and Place of the Study

This study was performed in the Ministry of Health Zekai Tahir Burak Women's Health Training and Research Hospital, Gynecologic Oncology Service between May 1 and September 1, 2010.

### 2.3. The Population and the Sample Group

The sample group was recruited by the calculation formula (58 patients). The researchers divided the sample group of 120 patients into one study and one control group, each comprising 60 patients. The patients in both groups had to have similar sociodemographic profiles; age and education, as well as same types of operation.

n = Z^2^S^2 ^/d ^2^

n = the number of the population

S^2 ^= the variable of the population from doing the pilot study of 20 patients

Z^2 ^= Derived from the opening mean of Z at the (1 - α)-100% validity level, α = .05 and Z = 1.96 hereby

d^2 ^= The mean of the discrepancy which is .05 hereby.

n = (1.96)^2^(.17)^2^/(.05) ^2^

n = 58 patients

#### Inclusion criteria

- Those without advanced cancer, diagnosed within the last 0-6 months, had not taken any chemotherapy or radiotherapy, between 18 and 65 years of age, literate, had not undergone any gynecologic cancer surgery, without visual/hearing/perception problems and willing and pleased to co-operate in this research.

### 2.4. Collecting Data

Approval of the hospital training, planning and coordination ethical committee was obtained for the implementation of the study. "Individual Information Form" and "State-Trait Anxiety Inventory" (STAI) were applied through face-to-face interview method before and after the procedure after explaining the purpose of the study to women who accepted to participate in the study and obtaining their written approvals.

The researchers listed the names of the patients admitted at the gynecology clinic and selected the ones with the eligible requirements. Randomly, one of each two patients were included in the study group while the other one was selected for the control group. The entire instruction program for the 60 patients in the study group was conducted by one single person, the researcher nurse working in the gyneoncology clinic and taking part in this study as a certified expert in the field. Instructions were given in the training room located within the clinic and lasted approximately an hour per patient.

The researcher introduced herself, informed, then, the patients on the objective of the research and asked for their co-operation by answering the questionnaires. State-Trait Anxiety Scale was applied to both groups at least one day prior to the operation while only State Anxiety Scale was re-applied to both groups before discharge after the operation. While informing the control group about the operation with routine information, the study group was informed in detail with the help of a written and visual 'patient information booklet'. Patients in the study group were given the written-visual information booklet during this instruction and received this instruction together with their primary care givers. This instruction process was realized in an interactive environment in which patients were able to ask questions concerning their states and get answers for these questions. Also, before discharge, an instruction assessment interview was carried out with these patients. Here, patients were asked whether they were satisfied with the instructions they received on their disease, on the stages and objectives of the operation, and on post-operative self-practices. All patients in the study group stated that they were adequately informed on the various aspects of their conditions and received satisfactory answers to their questions. It should also be noted that the patients in the control group were not subjected to any ethical inconvenience since they received routine nursing care, which includes a post-operation instruction period.

#### Patient Information Booklet

The booklet was an instructional tool giving information on gyneoncological surgery prepared by the researchers in the light of the literature on the subject. There were 3 teaching plans in the "Patient Information Booklet"; 1. the patients' pre-operative preparation. 2. the relaxation practice skills. 3. the post-operative self-practices at the clinic and at home. The booklet based on the systematic health instruction program consisted of contents and illustrations about:

• Locations of internal and external genital organs in the body, definition,

• The patients' pre-operative preparation (putting away valuable belongings, false teeth before being moved to the operating theatre, the emptying of the stomach and the intestines, the preparation of an operative skin, being given some medicine, such as a dose before bedtime, a muscle relaxant, a sedative, pre- medication and relaxation techniques (breathing relaxation, muscle relaxation, imagery).

• How to treat themselves after the operation, including information about the pain and discomfort of an operative wound, the length of the home recovery period, and the necessity of and the practices when coming for the post-operative appointment (HRT following surgical menopause and its effects and importance and the Kegel exercises and daily-life activities)

#### 2.4.1. Individual Information Form

This form consists of 15 items to determine demographic characteristics including age, occupation and educational status and characteristics related to the operation of the groups included in the study.

#### 2.4.2. State-Trait Anxiety Scale I-II

This scale is used in clinical applications and treatment to evaluate the anxiety levels of patients. The State-Trait Anxiety Inventory I-II, which was developed in 1970 by Spielberger and colleagues to evaluate the conditional and continuous anxiety levels separately, has been translated into Turkish by Oner and Le Compte and its validity and reliability for the Turkish Society has been evaluated [11]. The State-Trait Anxiety Inventory consists of two different scales with 40 items in total (each scale consists of 20 items). Scores exceeding 42 in the State-Trait Anxiety Inventory are considered as "high anxiety level".

### 2.5. Evaluation of the Data

Data obtained in the study were evaluated on the computer using SPSS package program. The following values and tests were used in the study:

1. The patients' personal data were calculated to find the average mean and the percentage and tested to find the difference by using the Chi-square

2. The anxiety-measuring form for the patient waiting for an operation was calculated to find the percentage, the mean and the frequency.

3. The pre-operative anxiety levels of the patients in the two groups, the study group and the control group, were compared through the use of the Independent t-test to find out the difference.

4. Variance analysis to see if there is a relationship between some characteristics of the participant and anxiety scores of patients.

## 3. Results

The researchers divided the sample groups into one study group and one control group, each consisting of 60 patients. No statistically significant differences were found between the distributions of age, educational status, marital status, children owning and income levels in the two groups (p > .05).

As seen in Table 1, 50% of the patients in the study group are in the 38-48 age group (study group mean age 48.52 ± 5.91, control group mean age 49.87 ± 6.21); 46.6% are graduates of primary school, 78.4% are housewives, income level of 65% is medium, 61.6% are married and 83.3% have children.

**Table 1:** Findings Related to the Socio-demographic Characteristics

Socio-demographic Characteristics	GROUPS				Total		Statistical Analysis*
				
	Study (n = 60)		Control (n =60)				
		
	n	%	n	%	n	%	
**Age**							

38-48	30	50.0	28	46.6	58	48.3	x ^2 ^= 0.593 p = 0.624
≥49	30	50.0	32	53.4	62	51.7	

**Educational Status**							

Literate	17	28.4	16	26.6	34	28.3	x ^2 ^= 0.738 p = 0.691
Primary School	28	46.6	30	50.0	57	47.5	
≥ High School	15	25.0	14	23.4	29	24.2	

**Marital Status**							

Married	37	61.6	34	56.6	71	59.1	x ^2 ^= 0.538 p = 0.464
Widow/divorced	23	38.4	26	43.4	49	40.9	

**Working status**							

Working	13	21.6	11	18.3	24	20.0	x ^2 ^= 0.018 p = 0.893
Housewife	47	78.4	49	81.7	96	80.0	

**Having children**							

Yes	50	83.3	43	71.6	93	77.5	x ^2 ^= 2.301 p = 0.129
No	10	16.7	17	28.4	27	22.5	

**Income status**							

Good	12	20.0	9	15.0	21	17.5	x ^2 ^= 2.114 p = 0.347
Medium	26	43.3	27	45.0	53	44.1	
Poor	22	36.7	24	40.0	46	38.4	

**Total**	60	50.0	60	50.0	120	100.0	

* Pearson chi-square and Fisher tests were used.

When medical characteristics are considered (Table 2), it was seen that 43.3% of the individuals in the study group had ovarian cancer, 46.7% was in Stage II, 76.7% underwent TAHBSO+PALND, 61.7% did not have a previous surgical experience. No statistically significant differences were found between the study and control groups as regards medical characteristics (p > .05).

**Table 2:** Findings Related to the Medical Characteristics of the Patients

Medical Characteristics	GROUPS				Total		Statistical Analysis*
				
	Study (n = 60)		Control (n = 60)				
		
	n	%	n	%	n	%	
**Diagnosis**							

Over Ca	26	43.3	21	35.0	47	39.2	x ^2 ^= 2.286 p = 0.319
Cervical Ca	13	21.7	15	25.0	28	23.3	
Endometrial Ca	21	35.0	24	40.0	45	37.5	

**Stage**							

Stage I	19	31.6	16	26.7	35	29.2	x ^2 ^= 0.843 p = 0.656
Stage II	28	46.7	25	41.7	53	44.2	
Stage III	13	21.7	19	31.6	32	26.6	

**Operation type**							

TAHBSO	20	33.3	24	40.0	44	36.7	x ^2 ^= 0.217 p = 0.642
TAHBSO+PALND	40	76.7	36	60.0	76	63.3	

**Operations history**							

Yes	23	38.3	27	45.0	50	41.6	x ^2 ^= 0.420 p = 0.517
No	37	61.7	33	55.0	70	58.4	

**Information about the disease**							

Yes	29	48.3	33	55.0	62	51.6	x ^2 ^= 0.391 p = 0.532
No	31	51.7	27	45.0	58	48.4	

**Total**	60	50.0	60	50.0	120	100.0	

* Pearson chi-square test was used.

It was noted during the admission period that 51.7% of the patients who were to become the study group later did not have adequate information on their disease. In addition, none of the patients in the two groups had had previous operation experience and none of them had been provided with the knowledge of how to reduce anxiety before.

In Table 3, the average preoperative state anxiety I-I in the study group was 63.43 ± 4.81, while it was 70.03 ± 6.18 in the control group was. The average postoperative state anxiety I-I in the study group was 62.98 ± 5.11, while the same for the control group was 69.65 ± 5.92. No statistically significant differences were found between the study and control groups (p > .05). While the average postoperative trait anxiety I-II levels of the patients in the study group was found as 66.83 ± 4.80, the control group was 71.45 ± 7.48. There was statistically significant differences between the two figures (p < .05).

**Table 3:** Pre- Postoperative Average Scores in STAI-I and Postoperative Average STAT-II Levels

TESTS	STAI-I Study X ± SS	STAI-I Control X ± SS	t	p*
**Preop**	63.43 ± 4.81	70.03 ± 6.18	-0.380	0.595
**Postop**	62.98 ± 5.11	69.65 ± 5.92	-0.263	0.728

**TESTS** **Postop**	**GROUPS**		**t**	**p**
			
	**Study** **X ± SS**	**Control** **X ± SS**		

**STAI-II**	66.83 ± 4.80	71.45 ± 7.48	4.311	0.004

* Student (independent sample) t test was used.

According to the assessment, the difference between the average state anxiety scores of the study and control group in pre- and postoperative periods according to socio-demographic characteristics given in Table 4 were not found to be statistically insignificant (p > .05).

**Table 4:** Comparison of the Average State Anxiety Scores in the Study Group According to Socio-demographic Characteristics

Socio-demographic Characteristics	Preop X±SS	Postop X±SS	p*
Age	64.42 ± 3.24	69.62 ± 4.97	p > .05
	
Educational Status	65.34 ± 2.82	66.51 ± 4.95	
	
Marital Status	64.75 ± 2.11	67.60 ± 4.71	
	
Working Status	66.45 ± 3.71	68.94 ± 4.82	
	
Economical Status	63.98 ± 2.97	70.50 ± 6.07	
	
Having Children	66.25 ± 3.54	68.73 ± 6.91	

* Variance analysis was used.

Our research revealed that the change in anxiety levels in the study group was inversely proportional to the patient's education and income levels while it was in direct proportion to the patient's age. However, the difference did not bear statistical significance (p > .05). While in the groups of married patients and patients with children the anxiety levels tended to decrease, the difference was again not of statistical significance (p > .05).

Considering the medical characteristics, no significant differences were seen between pre- and postoperative state anxiety levels in both groups (p > .05). Regarding the type of surgical procedures, there was evidence showing that the score of anxiety was higher for the patients undergoing TAHBSO+PALND surgery than for those undergoing only TAHBSO surgery. This was also valid for patients with advanced stages (p > .05).

## 4. Discussion

For most patients, admission to hospital for surgery can be very stressful. Studies in this area support that requirements of patients to be informed in the preoperative period are not met, and anxiety can arise from lack of information [8,12,13]. In this study, all the patients who did not have adequate information about their disease and operation (51.7% in the study group -before they were instructed- and 45% in the control group) stated that they wished to get information from the healthcare personnel. Emotional and psychological surgical preparation plays an important role in many areas of nursing.

In the study of Wade et al (2000) it was found that giving information could decrease anxiety, pain, as weel as post-operative complication. It was concluded in some studies that preoperative anxiety levels were high; however, the nursing approach and instructions given are effective in reducing the level of anxiety [14-16]. Ozdemir and Pasinlioglu (2009) found in their study on 34 study group cases and 32 control group cases undergoing hysterectomy with benign causes that while the average state anxiety score was 40.9 ± 6.3, it fell to 27.6 ± 3.7 in the postoperative period (p = 0.001). Average state anxiety score was found in the control group as 41.1 ± 7.8 in the preoperative period and as 40.4 ± 8.3 (p = 0.625).

Gallicchio et al (2005) in their interviews with 1142 patients undergoing hysterectomy in Maryland Institute for Women's Health, found that anxiety was experienced at a rate of 80% and the fear of not being able to get rid of cancer and the fear of impairment of the quality of life were particularly effective on anxiety. When other studies in this area are examined, it is seen that anxiety signs related to the uncertainty of the postoperative period were seen in 105 Chinese women who were to undergo hysterectomy; another study conducted in Pakistan in 2005 demonstrated that anxiety increased postoperatively in women who had inaccurate knowledge on hysterectomy [12,17]; in the study of Cardenas et al (2005) giving information through a written educational booklet to 30 patients who were planned to undergo hysterectomy reduced the frequency of post-operative anxiety, pain and other complications [18]; in the study of Beatrice and colleagues (2005), 65 patients who were to undergo hysterectomy experienced anxiety regarding potential pain and sexual problems in the postoperative period [11]. Other studies on psychological factors have shown that hysterectomy alone is not effective on anxiety [10,19,20]. Donoghue et al (2003) found anxiety with a rate of 29% in their study performed on 60 patients who has undergone hysterectomy. They found three months later that anxiety was still 22% [21]. Jawor et al (2001) found that women who had undergone hysterectomy experienced intense anxiety because of lack of information, reduction in self-respect, reduction in the quality of life and loss of social functions [22].

In our study, average state anxiety in the study group was found as 62.98 ± 5.11 in the preoperative period and as 63.43 ± 4.81 in the postoperative period. It was found that, compared to other studies, our results were higher. While the average postoperative trait anxiety I-II levels of the patients in the study group was found as 66.83 ± 4.80, the control group was 71.45 ± 7.48. In this study, we found that systematic preoperative instruction was effective on reducing the anxiety level (Table 3). The difference was found to be significant (p < .05). Therefore, the hypothesis has been accepted. Yen et al (2008) found that, among 68 patients who had undergone hysterectomy because of gynecologic cancer, anxiety was experienced at higher levels with those patients with sexual problems and with the ones experiencing deterioration in body image [23], Hemly et al (2008) found that anxiety signs were observed in 36.5% of 96 individuals who had undergone hysterectomy and this rate was 78.7% in nullipars [24], Ryan et al (1989) found in their study on 60 women in 35-55 age group who had undergone hysterectomy that the anxiety level, which was 55% in the preoperative period, fell to 31.7% in the postoperative period [25]; Lalinec and Engelsmann (1985) found in their study on 102 patients who had undergone hysterectomy because of gynecologic cancer that anxiety was rather high, and there was no difference between the pre- and postoperative anxiety levels [26]. In a study performed on 45 Nigerian women in 35-63 age group, anxiety was observed at a rate of 44.4% in the preoperative period while postoperative anxiety was found to be 68.4% [27]. Reis et al (2008) performed detailed interviews in 2006 to determine the views and beliefs of those undergoing abdominal hysterectomy (n = 31) under five headlines, namely "feminine identity", "relationships with the spouse - family", "sexual life", "menopause" and "relationships with relatives - social relationships", and it was stated that women experienced intense anxiety because they felt that the would lose sexual desire, their relationships with their spouses would be impaired, and they would not feel like a woman after surgical menopause [28]. In the study of Kantar and Sevil (2004), it was found that women experienced anxiety because "uterus is a very important organ for them", "loosing their uterus will reduce their self-confidence", "their sexual lives would end", "their spouses would not be interested in them anymore" and "their relationships would come to an end" [29].

The powerful social factors affecting the reactions of women after hysterectomy are indicated as the educational status, income level, cultural structure, age at hysterectomy, short decision period before the operation, little support from the spouse and existence of a mental disorder preoperatively [14]. In our study, no relationship was found between age groups and the level of anxiety (p > .05) (Tablo 4). While Gunaydin and Oflaz (1998) state that age does not affect the level of anxiety [30], which is a finding similar to ours, it was found in other studies that anxiety was experienced most profoundly in younger age groups [18,19,27,28].

Our research revealed that the change in anxiety levels in the study group was inversely proportional to the patient's education and income levels while it was in direct proportion to the patient's age. However, the difference did not bear statistical significance (p > .05). Similar studies demonstrated parallel results highlighting that there was no significant relationship between the educational status and level of anxiety [18,28,30]. While in the groups of married patients and patients with children the anxiety levels tended to decrease, the difference was again not of statistical significance (p > .05). This finding complies with results of other works asserting there is no significant difference between the sociodemographic features of women and anxiety scores [2-4,7-10].

Taking into consideration the medical characteristics, no significant difference was observed between pre- and postoperative state anxiety levels in both groups (p > .05). Regarding the type of surgical procedures, there was evidence showing that the score of anxiety was higher for the patients undergoing TAHBSO+PALND surgery than for those undergoing only TAHBSO surgery. This was also valid for patients with advanced stage (p > .05).

However, studies concluding that there is a significant difference between anxiety levels of patients according to the stage of their disease are not inexistent. In their study Montazeri et al (2003) state that the stage of the disease has an impact on the anxiety levels of the patients and that advanced-level patients experience higher anxiety levels compared to those of lower-level patients [15]. Another study shows that patients who underwent major surgeries tend to have higher anxiety levels than patients who had minor surgeries [8].

There are a number of relaxation techniques, such as relaxing the muscle, meditation, biofeedback, creating and imagination, taking a deep and rhythmic breath. All these techniques are simple, and take less time to practice. It will benefit the patients' physical state if they practice them regularly. When the relaxation techniques are added to the preoperative training program, there is a tendency to increase the nursing efficiency [10,19]. Research indicates that listening to music reduces anxiety scores, too [6]. In our study, preoperative information accompanied by relaxation techniques was associated with a reduction in anxiety levels.

## 5. Conclusion and Recommendations

In conclusion, the gyneoncologic patients who received preoperative instruction demonstrated lower anxiety levels of statistical significance when compared with patients who received only the routine nursing care. Therefore, our study suggests that nurses should receive training so as to integrate preoperative instructions into the routine nursing care. When relaxation techniques such as relaxing the muscles, taking deep and rhythmic breaths and involving care givers in care are added to preoperative instruction, there is a tendency to increase the efficiency of nursing and it is expected that these techniques will bring the emotional state of the patients back to normal conditions more rapidly.

In this regard this study provides a foundation for future clinical interventions to reduce post-surgery anxiety. It is recommended that information should be given verbally with written booklets and relaxation techniques.

## Limitations

The study subjects were limited only to the gyneoncologic surgery patients of Zekai Tahir Burak Hospital in the province of Ankara. The subjects might not be representative of all surgical patients.

## Competing interests

The authors declare that they have no competing interests.

## Authors' contributions

The work presented here was carried out in collaboration between all authors. GP, AK and TG defined the research theme. GP and AK designed methods and carried out the instructions, analyzed the data, interpreted the results and wrote the paper. TG co-discussed analyses, interpretation, and presentation. GP involved in drafting the manuscript. All authors have contributed to, seen and approved the final manuscript.
